# Does it take two to tango? RING domain self-association and activity in TRIM E3 ubiquitin ligases

**DOI:** 10.1042/BST20200383

**Published:** 2020-11-10

**Authors:** Filippo Fiorentini, Diego Esposito, Katrin Rittinger

**Affiliations:** Molecular Structure of Cell Signalling Laboratory, The Francis Crick Institute, 1 Midland Road, London NW1 1AT, U.K.

**Keywords:** protein structure, ring ligases, TRIM proteins, ubiquitin ligases

## Abstract

TRIM proteins form a protein family that is characterized by a conserved tripartite motif domain comprising a RING domain, one or two B-box domains and a coiled-coil region. Members of this large protein family are important regulators of numerous cellular functions including innate immune responses, transcriptional regulation and apoptosis. Key to their cellular role is their E3 ligase activity which is conferred by the RING domain. Self-association is an important characteristic of TRIM protein activity and is mediated by homodimerization via the coiled-coil region, and in some cases higher order association via additional domains of the tripartite motif. In many of the TRIM family proteins studied thus far, RING dimerization is an important prerequisite for E3 ligase enzymatic activity though the propensity of RING domains to dimerize differs significantly between different TRIMs and can be influenced by other regions of the protein.

## Introduction

An abundance of cellular functions is controlled by the attachment of a 76-residue ubiquitin (Ub) molecule to a protein substrate. Ubiquitination occurs via the combined action of three enzymes: an ATP-dependent ubiquitin-activating enzyme (E1) conveys the ubiquitin molecule onto a conjugating enzyme (E2), which in turn transfers it, in the case of RBR or HECT ligase enzymes (E3), first to the E3 or, in the case of ‘Really Interesting New Gene’ RING ligases, directly onto the substrate [1–3]. RING-type E3s constitute the largest family of E3 ligases of which the tripartite-motif (TRIM)-containing protein family, is a major subfamily. During the last decade, structural and mechanistic studies have made key contributions to providing a deeper understanding of the molecular details of RING E3 ligation activity and regulation [4,5]. In this review, we specifically discuss the role of the catalytic RING domain in TRIM ligase function and recent advancements in the understanding of the mechanistic details that lie at the basis of its ubiquitination activity.

## The RING domain and TRIM E3 ligases

**Figure 1.**
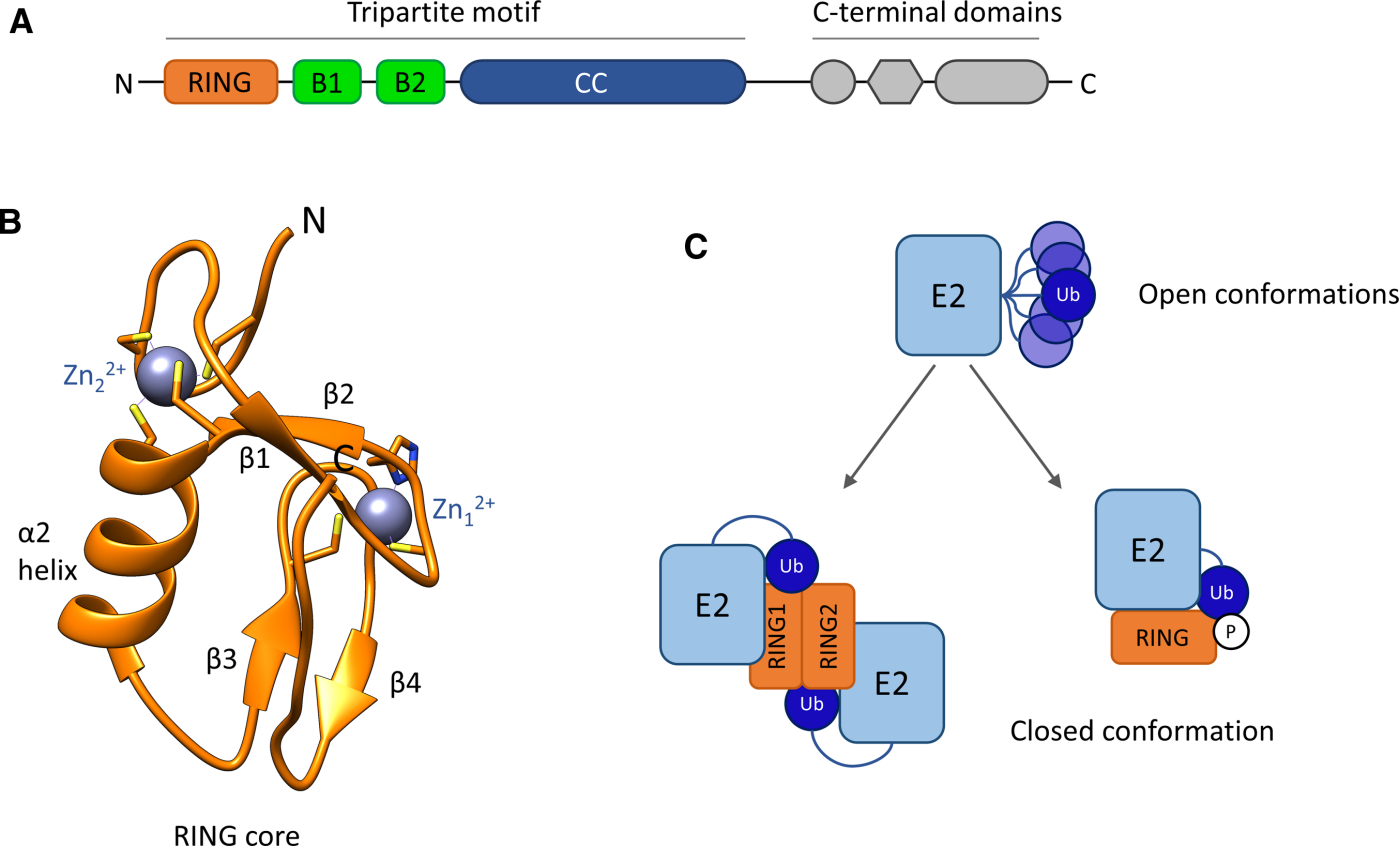
TRIM protein domain composition with details of RING domain structure and function. (**A**) Schematic representation of the domain composition of TRIM family proteins. (**B**) Canonical structure of a core RING domain (PDB: 5FER) with secondary structural elements and Zn^2+^-binding sites indicated. (**C**) Schematic depicting the multiple conformational states of E2∼Ub conjugates: the ‘open conformation’ and the ‘closed conformation’ formed upon interaction with RING domains in which ubiquitin contacts the E2 and the thioester bond is primed for ubiquitin transfer.

The TRIM ligase family contains over 70 members, which, with a few exceptions, are characterized by a common architecture of an N-terminal tripartite motif (also referred to as RBCC) comprising a RING domain followed by one or two zinc-coordinating domains called B-box1 and B-box2 and a long coiled-coil (CC) region (Figure 1A) [6–10]. The coiled-coil domain of TRIM proteins mediates homodimerization and possibly higher-order oligomerization. All X-ray structures of TRIM CCs determined so far show an anti-parallel arrangement for the homodimer, suggesting a common overall architecture of TRIMs where the catalytic RING domain is placed on opposite sides of its rod-like central CC domain [11–14]. In most members of the TRIM family, the tripartite motif is followed by a C-terminal region with varying domain composition that generally mediates substrate recognition and is used to classify TRIM proteins into 11 different individual classes (Figure 1A) [15].

The RING core is a small domain of 40–70 residues found in proteins from plants to humans. A cross-braced pattern of conserved cysteine and histidine residues coordinating two zinc ions maintain the native fold (Figure 1B) [16,17]. The RING domain acts as a matchmaker bringing the E2∼Ub conjugate (∼ denotes a thioester bond) into close proximity of the substrate bound via C-terminally located substrate-recognition domains in TRIM proteins. This mechanism of catalysis does not rely on a classical active site, and instead promotes ubiquitin transfer from the E2∼Ub conjugate to a lysine residue of the substrate by stabilizing an E2∼Ub conformation in which the E2 contacts the I44 hydrophobic patch of ubiquitin, referred to as the ‘closed conformation’ (Figure 1C) [18–21]. In the unbound state, E2∼Ub conjugates often populate an ensemble of extended ‘open’ states, in which reactivity towards lysine residues is low [22,23]. In the closed conformation, however, the RING domain holds the thioester bond between ubiquitin and the E2 active site cysteine in a geometry ‘primed’ for nucleophilic attack by a substrate lysine residue. Stabilization of the closed state can be achieved in different ways (Figure 1C): in monomeric RINGs, structural features outside the core RING, such as a phosphorylated tyrosine residue (Y363) in the case of Cbl-b (PDB: 3ZNI), synergize with the RING domain to capture the closed E2∼Ub form [24]. In contrast, several RING-type E3s including many TRIM ligases stabilize the closed E2∼Ub conformation through RING dimerization, where each protomer contacts an E2 whilst each of the conjugated ubiquitin molecules makes simultaneous contacts with both RING molecules [18]. This cooperative mechanism of ubiquitin binding involving both RING domains explains why dimerization is essential for the activity of these RING ligases. Accordingly, the increasing number of available structures of TRIM RING domains in isolation, in complex with E2s or E2∼Ub conjugates, and in the context of larger TRIM fragments (Table 1) adhere to a pattern whereby RING dimerization is the prevalent requirement conferring E3 activity, though exceptions exist [25–31].

**Table 1.** Structural information available on TRIM RING domains

TRIM	PDB IDs
TRIM5α	4TKP (RING:UBE2N);
TRIM19	5YUF (RING tetramer); *2MWX (RING monomer)*
TRIM21	5OLM (RING:B-box2); 6S53 (RING:UBE2N-Ub); 6FGA (RING: UBE2E1);
TRIM23	5VZV (RING); 5VZW (RING:UBE2D2-Ub).
TRIM25	5FER (RING:UBE2D1-Ub); 5EYA (RING: UBE2N-Ub)
TRIM28	6QAJ (RBCC); 6QU1 (RBCC:SMARCAD1 CUE1); 6H3A (RBCC:SMARCAD1 CUE1); *6I9H (RING)*
TRIM32	5FEY (RING); *2CT2 (RING)*
TRIM37	3LRQ (RING)
TRIM56	5JW7 (RING-SopA)
TRIM69	6YXE (RING)

Structures in italic have been solved by NMR spectroscopy, all others by X-ray crystallography.

## Structural features of RING dimerization

**Figure 2.**
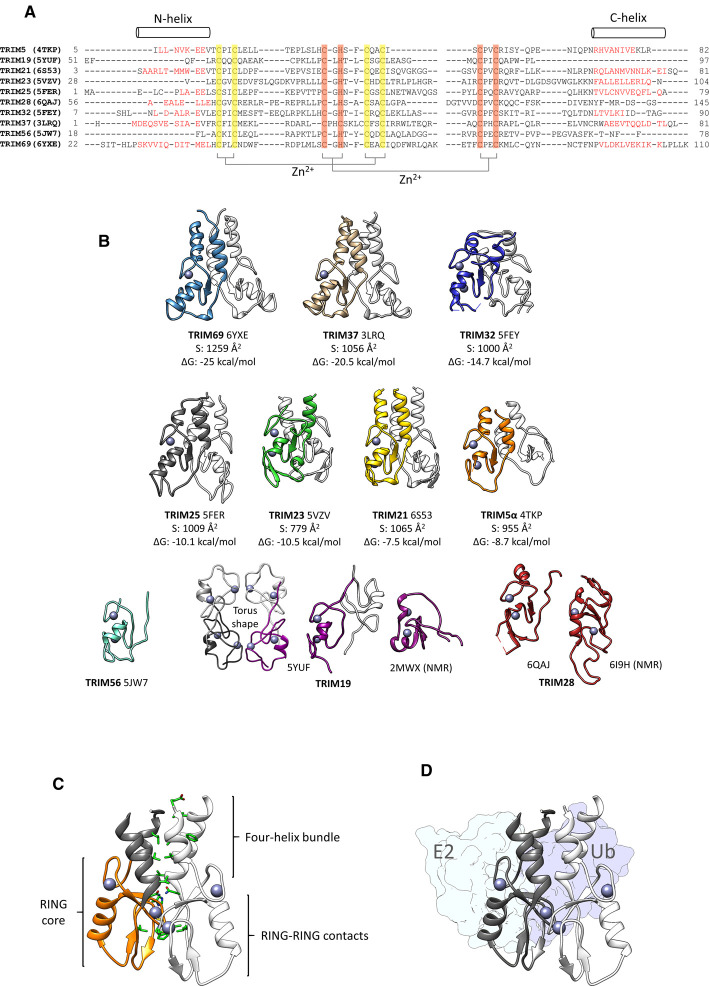
Structures of TRIM RING domains. (**A**) Structure-based sequence alignment of available TRIM RING crystal structures produced by the webserver SALIGN [53]. Only the residues for which electron density is seen are included in the alignment. The Cys and His residues coordinating the Zn^2+^ atoms are highlighted and the position of the N- and C-terminal helices mediating dimerization are indicated. (**B**) Comparison of available structures of TRIM RING domains. All RING domains are displayed in the same orientation. Zinc atoms are coloured in grey. For TRIM19 the torus-shaped tetramer seen in the crystal structure is also shown. Buried surface area and solvation free energy gain upon dimerization are listed. (**C**) Details of the TRIM25 RING dimer interface highlighting the residues (green) in hydrophobic interactions between the N-and C-terminal α-helices and those in the RING cores interface where one of the core monomers is depicted in orange. (**D**) The TRIM25 dimer with an E2∼Ub conjugate bound to the dark grey monomer. The E2∼Ub conjugate bound to the monomer coloured in white has been omitted for clarity.

In all TRIM family RINGs that have been shown to self-associate, but TRIM19, RING dimerization is achieved via hydrophobic interactions between aliphatic side chains of residues present in short α-helical segments at the N- and C-termini of the zinc-coordinating core RING domain that form a 4-helix bundle. Additional contacts contributed by the core RING domain, mainly from residues in the loop connecting ß-sheets ß1 and ß2 stabilise the dimer interface (Figures 1B and 2A–C) [25,26]. While the majority of crystal structures of TRIM RING domains show them to form symmetric dimers, their oligomeric state in solution varies dramatically and spans from constitutively dimeric to monomeric, with others existing in a monomer-dimer equilibrium, that is shifted towards a dimeric species by an increase in concentration or binding of a Ub-loaded E2 (Figure 2D). In contrast, the RING of TRIM19 (Figure 2B) which is involved in the formation of nuclear bodies forms a torus-shaped tetramer (described in detail below) [32]. In the following paragraphs we will provide an overview of these different behaviours, present those that are strictly monomeric and discuss the possible reasons behind this variation in self-association and how it may affect catalytic activity.

At present only two RING domains, from TRIM32 and TRIM69, have been shown by biophysical techniques to form a constitutive dimer in solution [25,31]. Analysis of all available crystal structures of dimeric TRIMs suggests that the solvation free energy gain upon dimerization might be a useful metric to predict whether a TRIM RING domain will form a strong dimer in solution [31,33]. Accordingly, TRIM32 and TRIM69 have the largest gains of −14.7 kcal/mol and −25 kcal/mol, respectively, of those RING structures that have also been characterized in solution. Based upon this metric, TRIM37 (−20.5 kcal/mol, PDB: 3LRQ) is predicted to form a constitutive dimer as well, but biophysical studies describing its solution behaviour are required to support this notion. The ability of the TRIM32 RING domain to self-associate is both necessary and sufficient for its catalytic activity: the deletion of the N- and C-terminal helices produces a monomeric form of the protein, whose NMR solution structure has been determined (PDB: 2CT2), and which lacks any detectable E3 ligase activity [25]. Similarly, a dimerization-disrupting mutation I85R in the 4-helix bundle, also impedes ubiquitin discharge from a UBED3-Ub conjugate and UBE2N/UBE2V1 driven K63-linked polyubiquitin chain formation.

In contrast to TRIM32 and TRIM69, other TRIM RING domains analysed thus far exist in a monomer-dimer equilibrium in solution, which in the case of TRIM25 is strongly shifted towards the monomer, with dimerization only observable by NMR as protein concentration increases [25]. In contrast, the TRIM23 RING domain shows a propensity to dimerize at protein concentrations as low as 100 µM [28]. The RING domain of TRIM5α has been described as monomeric in solution based on velocity sedimentation experiments at protein concentrations up to 2 mg/ml (∼200 µM) [26]. Nevertheless, RING self-association is crucial for activity as mutations in the dimer interface of TRIM5α (I77R), similar to TRIM25 (V72R) [25], interfere with catalytic activity while tandem RING constructs of either TRIM display significantly enhanced activity. Interestingly, in TRIM25 activity of the RBCC is higher than that of the isolated RING, observed with both UBED3 and UBE2N/UBEV1, suggesting that the presence of the CC domain promotes RING dimerization.

## RING dimerization in full-length TRIMs

**Figure 3.**
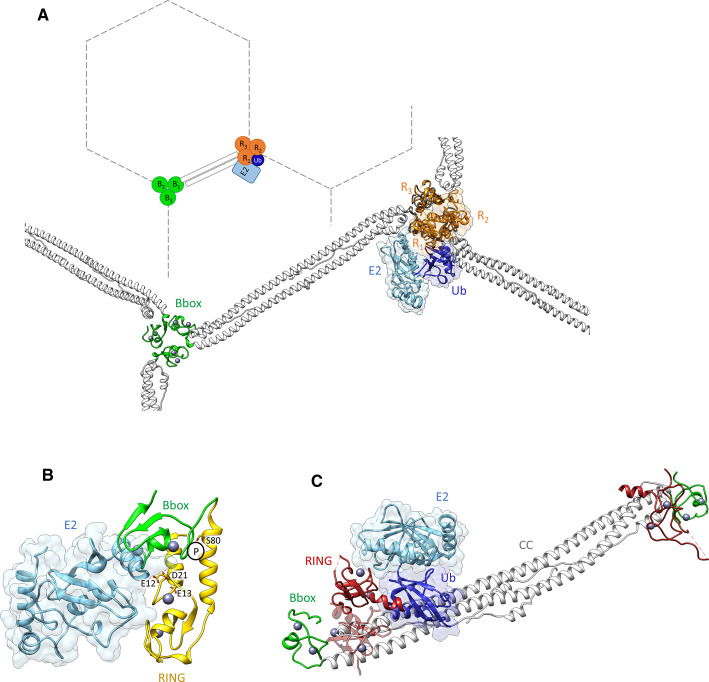
TRIM RING domains in the context of larger protein constructs. (**A**) Model of TRIM5α RING dimerization triggered by B-box-mediated trimeric assembly. The model was created using the following PDB files: 4TKP, 5W9A, and 5IEA. RING domains are coloured in orange, B-box domains in green, CC domains in white, E2 in cyan, and Ub in blue. The functional RING dimer at the three-fold interface (R1, R2) is highlighted by surface representation. For clarity only one copy of the E2∼Ub conjugate, bound to R1, is shown. (**B**) Superposition of the structure of the TRIM21 RING (yellow) and B-box (grey) domains (PDB: 5OLM) with the TRIM21 RING in complex with UBE2N-Ub (blue), showing that UBE2N and the B-box are structurally competitive. Phosphorylation at residue S80, located at the centre of the RING/B-box interface, displaces the B-box domain allowing E2 recruitment. Residues forming the tri-ionic anchor are highlighted. (**C**) Structure of the TRIM28 RBCC domain. RING domains are coloured in dark red, B-boxes in green, the CC in white, the E2 in cyan, and the Ub in blue. The E3/E2-Ub complex was obtained by superimposing the TRIM28 RBCC structure (PDB: 6QAJ) with the structure of the TRIM23 RING in complex with E2-Ub (PDB: 5VZW). The picture highlights the structural competition between the E2-Ub bound to the RING domain and the CC domain. A second RING protomer, displayed in transparent dark red, forming a putative functional dimer, would clash with the CC domain.

The anti-parallel nature of the CC domain places the two RINGs at opposite ends of a given TRIM homodimer implying that RING dimerization and catalytic activity will require association of two or more TRIM homodimers into a higher-order assembly, or alternatively occur within one homodimer. In fact, the ability to form tailored higher-order assemblies is key to the activity of some TRIMs, best understood in the case of TRIM5α, a retroviral restriction factor that targets the viral capsid core and synthesizes K63-linked polyubiquitin chains to activate innate immune signalling pathways. A range of biochemical and biophysical studies have analysed the self-association properties of individual domains of TRIM5α, and combined with cellular studies, shed light on the mechanism behind higher-order self-assembly and dimerization of the RING domains [34–37]. These studies suggested that the B-box domain makes important contributions to higher order assembly, which however were difficult to quantify as the isolated B-box tends to aggregate in the absence of the adjacent CC that protects a hydrophobic surface. To circumvent these problems two groups independently designed ‘mini-TRIMs’ in which portions of the opposing monomers of the CC are connected by a short linker to produce a construct that contains a single B-box [38,39]. Elegant studies using these mini-TRIMs showed that the B-box domain of TRIM5α trimerizes, which together with CC-mediated anti-parallel dimerization [11,13] leads to formation of a hexagonal lattice with the B-box at the centre of the trimer (Figure 3A). This arrangement matches the lattice symmetry of the capsid and brings the RING domains into close proximity to allow dimer formation. Furthermore, the templated hexameric assembly of TRIM5α on the capsid switches the ligase activity from N-terminal Ube2W-mediated auto-monoubiquitination, which triggers TRIM5α turnover, to anchored K63-linked chain formation which induces immune signalling pathways and virus destruction [40,41]. At present it is not understood what the role of the third RING domain in this hexagonal arrangement might be: it has been suggested that it may act as a substrate for auto-ubiquitination, though further studies are required to fully understand its function. In summary, in the case of TRIM5α higher order assembly is mediated by 2 structural elements: CC-mediated dimerization and B-box2-mediated trimerization and serves to couple substrate recognition with activation of catalytic E3 ligase activity.

## Regulation of TRIM ubiquitination activity

Many of the TRIM proteins studied thus far are constitutively active when overexpressed in cells or studied *in vitro*. However, in a physiological setting, activity might possibly be regulated by recognition of substrate and cognate E2s, or through additional stimuli such as post-translational modifications. The first example of such regulation has recently been identified in TRIM21, a cytosolic antibody receptor [42], which is kept in an inactive form through auto-inhibitory interactions. Activation of TRIM21 initiates autoubiquitination on its N-terminus, which is then extended with K63-linked polyubiquitin chains [43]. Biochemical experiments, combined with the crystal structure of a RING-B-box2 construct of TRIM21 (PDB: 5OLM) revealed that the B-box occupies the E2 binding site of the RING, an interaction stabilised by electrostatic interactions, and acts as an E2 mimic (Figure 3B) [44]. Removal of B-box-mediated autoinhibition is achieved by phosphorylation of S80 located in a variant *p*LxxIS (*p* is hydrophilic) motif that is recognized by IKKß and TBK1 kinases. Phosphorylation of S80 displaces the B-box, thereby allowing E2 recruitment to restore TRIM21 ubiquitination activity. Similar to other TRIM RING/E2∼Ub complexes, the crystal structures of TRIM21 with its cognate E2s, UBE2E1 (PDB: 6FGA) [30] and a UBE2N-Ub conjugate (PDB: 6S53) [29], show symmetrical complexes surrounding a RING dimer, while in solution the RING is in a monomer-dimer equilibrium. Nevertheless, mutants that impair dimerization (M10E and M10E/M72E) remain active and able to catalyse unanchored K63-linked ubiquitin chain synthesis, UBE2W-primed anchored K63 chain extension and ubiquitin discharge, however less efficiently than the wild-type protein, suggesting that E3 activity may not strictly require a dimeric RING. A possible explanation for the observed monomeric RING activity is that residue E13 - corresponding to residue E10 in TRIM25 [25], which has been shown to be critical to contact ubiquitin - allows a monomeric RING of TRIM21 to contact both E2 and bound ubiquitin simultaneously, obviating the requirement for dimerization. Consistent with this hypothesis, E13R was shown to reduce TRIM21 catalysis. Moreover, in combination with residues E12 and D21, E13 forms a tri-ionic motif (Figure 3B) which provides conformation-specific anchor points that allow an UBE2N∼Ub molecule to be wrapped around the RING, thereby locking the closed conformation and promoting ubiquitin discharge (PDB: 6S53) [29]. Being located in one RING monomer and critical for the capture of the E2∼Ub complex, the identification of the tri-ionic motif could explain why monomeric TRIM21 RING seems capable of performing catalysis. This mechanism of E3 ligase activity, apparently needless of ‘canonical’ activation through RING dimerization, is instead regulated by an autoinhibitory mechanism involving the B-box of TRIM21 and TRIM phosphorylation [44].

## TRIM ligases with monomeric RINGs

The case of TRIM21 hints at other levels of regulation for those TRIM members which do not show constitutive ubiquitination activity. One such example is represented by Class VI TRIM proteins, for which the mechanism behind the observed cellular E3 ligase activity has remained elusive. This class of TRIMs - which includes TRIM24, TRIM28 (KAP1) and TRIM33 - is best known for its role as transcriptional regulators, a role which appears functionally unrelated to their ubiquitination activity [45,46]. Intriguingly, the isolated RING domains of TRIM24, TRIM28, and TRIM33 were shown to be unable to promote ubiquitin discharge from E2∼Ub conjugates [47]. Similarly, no E3 ligase activity was detected for full-length TRIM28, excluding the possibility that other flanking structural elements, like the B-box2 domain for TRIM5α or the coiled-coil, are required to promote E3 function. Biophysical analysis of the isolated RINGs of TRIM24, TRIM28, and TRIM33 revealed that these proteins are monomeric in isolation with no concentration-dependent oligomerization and, contrary to other TRIMs, dimerization could neither be promoted by addition of an E2∼Ub conjugate nor by fusing two RING domains via a short linker as has been done in other TRIMs. The TRIM28 RING NMR solution structure (PDB: 6I9H) revealed that TRIM28 shares the canonical RING domain fold, but, whilst showing a modest propensity for an N-terminal helical secondary structure, it lacks the C-terminal α-helix characteristic of dimeric TRIM RING domains (Figure 2) [47]. The C-terminus is largely unstructured and highly dynamic, precluding dimerization in the same fashion as other TRIM proteins. Furthermore, no interaction could be detected between the RING of TRIM28 and the ubiquitin conjugate of UBE2D, a promiscuous E2 that shows activity with most E3 ligases. A possible reason for this observation may be the lack of a conserved hydrophobic residue (L17 in TRIM25) at the interface between the RING domain and the E2, which is replaced by an arginine residue in TRIM28. However, mutation of the TRIM28 arginine residue to the leucine residue present in TRIM25 (R69L) did not prove to be enough to rescue activity.

A recent biochemical analysis of a TRIM28 RBCC fragment highlighted a high propensity for oligomerization - dimers can self-assemble into tetramers, octamers, and possibly higher-order species at high local concentrations, primarily via B-box1-mediated interactions [48]. This capability to form higher-order assemblies could not be linked to its role in transcriptional silencing, while its effect on ubiquitination activity was not tested. Moreover, the crystal structure of TRIM28 RBCC (PDB: 6QAJ), fused N-terminally to T4 lysozyme to improve crystal quality, is incompatible with RING dimerization, as the formation of RING dimers, as well as binding of E2∼Ub conjugate, would be structurally competitive with the CC domain (Figure 3C). Equally, the TRIM28 RINGs do not form significant crystal contacts with each other which could hint to activation via higher-order oligomer formation. The lack of RING dimerization and E2 binding was therefore proposed to be responsible for the absence of observable catalytic activity of TRIM28. Nevertheless, E3 ligase activity of Class VI TRIMs has been reported in cellular assays: this activity could be explained by the presence of cellular binding partners such as MAGE proteins, which bind TRIM28 and promote ubiquitination of p53 and AMPK to induce their degradation [49,50], or possibly by post-translational modifications. Alternatively, class VI TRIM proteins may constitute inactive RING ligases that require heterodimerization with active RING partners for physiological activity.

The only other structure displaying a monomeric TRIM RING domain is the crystal structure of the human TRIM56 RING domain bound to the Salmonella effector SopA (PDB: 5JW7), a bacterial HECT-like E3 ligase [51]. The structure reveals a monomeric zinc-binding core RING domain with an overall structure similar to that of other TRIMs but lacking the N- and C-terminal sequences commonly responsible for RING dimerization (Figure 2B). However, the absence of dimerization of the TRIM56 RING in this complex might be the result of the C-terminal fusion of SopA to the RING domain for the purpose of crystallization. Indeed, structural alignment of the TRIM32 dimer with TRIM56 RING in the SopA/TRIM56 complex structure revealed only minor clashes between the TRIM32 dimer and SopA, indicating that SopA interaction may be compatible with TRIM56 dimerization in solution. Instead, the Salmonella effector was shown to inhibit TRIM56 activity by occluding its E2-binding site. Further studies addressing self-association of TRIM56 are necessary to clarify if its RING domain can indeed function in a monomeric form.

## TRIMs with unusual oligomeric RINGs

An outlier with respect to RING oligomerization is TRIM19 (PML), which forms a ‘torus-shaped’ tetramer. Despite being described as monomeric in solution by an NMR study (PDB: 2MWX), it crystallizes in a tetrameric form (PDB: 5YUF) via a pattern of conserved TRIM19-specific residues (Figure 2B) [32,52]. Tetramerization is suggested to be a requisite for its observed sumoylation activity and formation of nuclear bodies and involves residues that are highly conserved amongst TRIM19 orthologs. Tetramerization is mediated via multiple interfaces: the first is formed by hydrophobic interactions between the side chain of F52 in one subunit and leucine residues (L70 and L81) together with the W95 side chain in the other. On the opposite side, the aromatic ring of F54 is sandwiched between the side chains of two lysine residues, K65 from the same subunit and K68 from another. Furthermore, two subunits come together by ‘hand-shake-like’ hydrophobic interactions involving either leucine residues L73. Biophysical and biochemical studies showed that, in solution, the RING of TRIM19 appears to be in equilibrium between monomeric, dimeric and tetrameric species. Interestingly, whilst mutation of L73 into glutamate disrupts tetramerization, unexpectedly, residues at the C-terminus of the RING domain (N106 and L112) - corresponding to the helical region important in the dimerization mechanism for other RINGs and not included in the construct used for X-ray crystallography - not only appear to disrupt tetramerization but also have an effect on the nuclear bodies biogenesis and TRIM19 sumoylation activity. At this point it is not clear why the RING of TRIM19 requires tetramerization for catalytic activity, and it will be interesting to see how tetramerization manifests itself in the full-length protein.

## Conclusions

Dimerization of the RING domain, whether entirely self-promoted or supported by other elements within the full-length protein, appears to be a common strategy for conferring E3 ligase catalytic activity to TRIM family proteins. The ability to form helical segments N- and C-terminal to the core RING is imprinted in many TRIM primary sequences (Supplementary Figure S1) and appears to be a prerequisite for the formation of dimers. Nevertheless, while some TRIMs form constitutive RING dimers, in others these N- and C-terminal segments, which contain the aliphatic residues that stabilize the core of the four-helix bundle, might be in dynamic exchange in solution between helical and random-coil secondary structures. A shift of the equilibrium towards a stable dimer may only occur as the local RING concentration increases - for instance, following the binding of the E2∼Ub conjugate, which could be a regulated event. In the context of the full-length protein, where the RING domains are located at either end of a rigid anti-parallel coiled-coil, RING dimerization likely requires additional protein interactions mediated by other structural elements or the formation of higher-order assemblies, potentially driven by substrate-binding.

The functional requirements underlying these differences in behaviour are not clear at this time and we speculate that the strength of RING self-association and need for other supporting elements might be a means to regulate overall catalytic activity. Detailed studies on the full-length proteins are urgently required to understand the key factors dictating the relationship between the overall oligomeric state and the formation of catalytically active RING dimers in TRIM ligases, and cryo-electron microscopy will likely play an important role in providing a molecular description of these factors.

## Perspective

- *Importance of the field*: TRIM E3 ubiquitin ligases are crucial regulators of many cellular processes and their dysfunction is associated with a variety of pathological conditions. Insight into the molecular features driving catalytic activity is crucial to understanding their physiological function.
- *Summary of current thinking*: Self-association of TRIM ligases is known to be important for their function yet the precise details of the link between ligase activity and oligomeric state remain largely unknown. Dimerization of the catalytic RING domain is important for ubiquitination activity in most TRIM proteins studied so far.
- *Comment on future directions*: The future availability of structures of full-length TRIM proteins, bound to E2-Ub conjugates and substrates, will have a major impact on our understanding of the function, catalytic activity and regulation of this protein family.

## Open Access

Open access for this article was enabled by the participation of The Francis Crick Institute in an all-inclusive *Read & Publish* pilot with Portland Press and the Biochemical Society under a transformative agreement with JISC.

## Abbreviations

CC: coiled-coil
TRIM: tripartite-motif
