# Supplementary material for: Does it take two to tango? RING domain self-association and activity in TRIM E3 ubiquitin ligases
Source: Biochem Soc Trans. 2020 Nov 10;48(6):2615–24. doi: 10.1042/BST20200383 (PMC7752041; doi:10.1042/BST20200383)
Supplement: Supplementary Figure S1 [file BST-48-2615-s1.pdf]

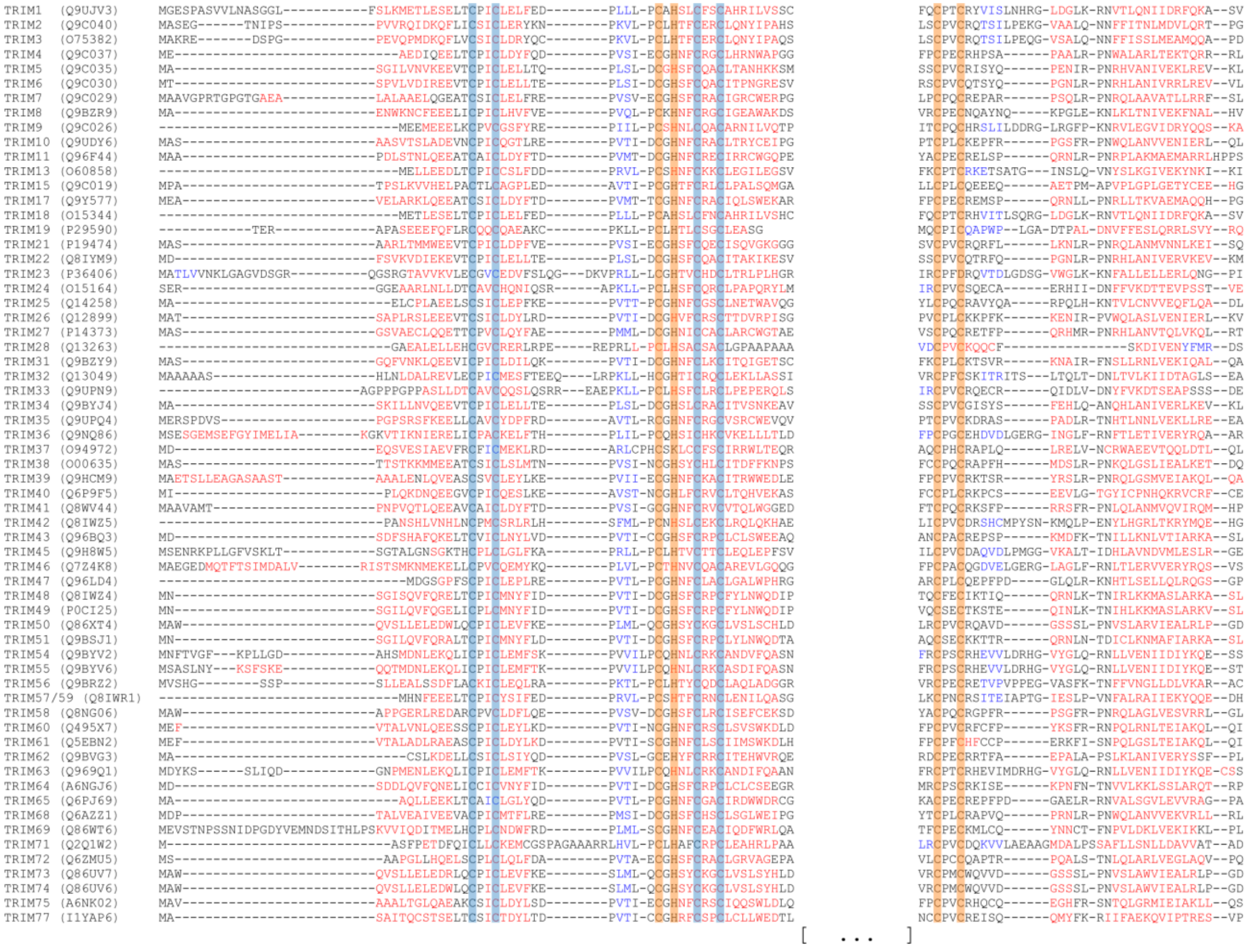

Figure S1. Alignment of TRIM family RING domains

TRIM family RING domain alignment based on secondary structure prediction and available structural coordinates performed by PROMALS3D (52). Residues coloured in red and blue represents amino acids either observed in, or with a propensity to form, α-helical and β-strand conformations, respectively.
